# Vitamin - D deficiency as a risk factor in primary hypertension - A cross sectional study

**DOI:** 10.6026/973206300211248

**Published:** 2025-05-31

**Authors:** Pushpendra Singh Sengar, Amit Saxena, Anurag Jain

**Affiliations:** 1 Department of Medicine, Bundelkhand Medical College and Hospital, Sagar, Madhya Pradesh, India

**Keywords:** Vitamin D deficiency, primary hypertension, cardiovascular risk factors, serum 25(OH) D levels, blood pressure, hypertension pathophysiology

## Abstract

The level of serum vitamin - D among patient with essential hypertension is of interest. Vitamin D plays a significant role in cardiovascular health, with emerging evidence linking its deficiency to hypertension. Therefore, it is of interest to assess the association between vitamin D deficiency and primary hypertension. A total of 100 patients with primary hypertension were evaluated for serum 25(OH)D levels. A significant proportion of hypertensive patients were found to have low vitamin D levels. These findings suggest that vitamin D deficiency may be a potential modifiable risk factor in the management of primary hypertension.

## Background:

In addition to its well-established role in calcium and bone metabolism, vitamin - D deficiency has been linked to a range of chronic diseases, including metabolic syndrome, hypertension, diabetes mellitus, coronary artery disease and heart failure [1]. Among these, hypertension is recognized as the leading preventable cause of premature death and disability worldwide [2]. Multiple etiological factors contribute to hypertension, such as age, race, family history, obesity, a sedentary lifestyle, tobacco use, high salt intake, psychological stress and excessive alcohol consumption. Vitamin - D deficiency is also considered one of these contributing factors, with studies suggesting an inverse relationship between vitamin - D levels and both blood pressure (BP) and the incidence of hypertension. However, while a few studies have demonstrated the blood pressure-lowering effects of vitamin - D supplementation, the majority have not observed a significant benefit [3]. This inconsistency in findings may be attributed to variations in study populations, baseline vitamin - D levels, dosage and duration of supplementation and the presence of comorbid conditions. Pre-hypertension, the clinical precursor to hypertension is a critical risk factor for the progression to full-blown hypertension as well as for the development of cardiovascular diseases [4]. Early intervention and primary prevention are key strategies for delaying or preventing the onset of hypertension. Identifying modifiable risk factors, such as vitamin - D deficiency, is crucial in developing cost-effective preventive strategies, especially in resource-limited settings. Emerging evidence indicates that vitamin - D plays a significant role in the pathogenesis of arterial hypertension. Animal models lacking vitamin - D receptors exhibit both arterial hypertension and increased renin expression [5, 6-7]. This suggests that vitamin - D modulates blood pressure primarily through its regulatory effect on the renin-angiotensin-aldosterone system (RAAS) [6]. Therefore, it is of interest to assess the association between vitamin D deficiency and primary hypertension.

## Materials and Methods:

## Study site:

This study is conducted in Department of general Medicine Bundelkhand Medical College Sagar Madhya Pradesh.

[1] Study population: All the eligible hypertensive patients presenting to Medicine OPD at our centre.

[2] Study design: Cross- sectional observational study.

[3] Sample size and study duration: This study consisted of 100 individuals who presented to our hospital's outpatient clinic between 1 May 2023 and 1 Feb 2024 for routine health check-up.

## Inclusion criteria:

[1] Patients with essential hypertension.

[2] Patients between age group of 25 years to 60 years.

[3] Both sexes were included.

## Exclusion criteria:

[1] Individuals below 25 years and above 60 years of age were excluded.

[2] Patients with renal failure.

[3] Pregnancy.

[4] Patients with secondary hypertension.

[5] Patients who received vitamin - D treatment in the last 6 months.

## Methodology:

For this study, a total of 100 patients were selected using a simple random sampling method from individuals attending the general medicine outpatient department. Informed consent was obtained from all participants prior to enrollment. Patients with secondary hypertension, chronic kidney disease, liver disorders, or those on vitamin - D supplementation were excluded to minimize confounding factors. Blood pressure (BP) was measured twice for each participant using a calibrated aneroid sphygmomanometer. Measurements were taken from the right arm while participants were seated, after a minimum rest period of five minutes in a quiet environment. Care was taken to use the appropriate cuff size for accurate readings. The two BP measurements were taken at an interval of at least two minutes and the average of the two readings was used for analysis. All BP values were recorded in millimeters of mercury (mmHg). Hypertension was defined according to the 2018 European Society of Cardiology (ESC) and European Society of Hypertension (ESH) guidelines, as a systolic BP ≥ 140 mmHg and/or a diastolic BP ≥ 90 mmHg. In addition to BP measurement, demographic and clinical data such as age, gender, body mass index (BMI), lifestyle factors (*e.g.*, smoking, physical activity) and history of comorbidities were recorded using a structured questionnaire. Blood samples were collected for the analysis of serum 25-hydroxyvitamin - D [25 (OH) D] levels, which were measured using a chemiluminescence immunoassay method in a certified laboratory.

## Statistical methods:

Descriptive analysis was made using frequency and proportion for categorical variables and mean, standard deviation for continuous variables. The mean values were analysed between and within the study groups using Independent t test. Categorical outcomes were analysed between study groups using the Chi-square test. P-value < 0.05 was considered statistically significant. IBM SPSS was used for statistical analysis.

## Results:

In this study, 100 hypertensive patients were included. The gender distribution showed that 72% of the participants were male (n = 72), while 28% were female (n = 28). The mean height of participants was 163.1 cm (SD = 6.4 cm) and the mean weight was 65.9 kg (SD = 8.5 kg). The mean BMI was 25.1 (SD = 3.07), which is classified as overweight according to standard BMI categories. When examining blood pressure, both systolic BP and diastolic BP were significantly higher in the hypertensive group, with mean systolic BP of 163 mmHg (SD = 16.7) and mean diastolic BP of 95 mmHg (SD = 9.3). Both systolic and diastolic BP values showed a statistically significant difference (p = 0.001), confirming that the participants met the criteria for hypertension, defined as systolic BP ≥ 140 mmHg and/or diastolic BP ≥ 90 mmHg according to the 2018 ESC guidelines. Vitamin - D Levels in Hypertensive Patients. The distribution of serum vitamin - D levels among the hypertensive participants revealed a high prevalence of vitamin - D deficiency. Of the 100 patients, 58 patients (58%) had serum vitamin - D levels less than 37.5 nmol/L (Level I), indicating severe deficiency. 7 patients (7%) had vitamin - D levels between 37.5 and 49.9 nmol/L (Level II), while 20 patients (20%) had levels ranging from 50 to 74.9 nmol/L (Level III). A smaller proportion, 15 patients (15%), had serum vitamin - D levels greater than 75 nmol/L (Level IV) and which is considered sufficient. The distribution of vitamin - D levels was found to be statistically significant (p = 0.007), suggesting an association between low vitamin - D levels and essential hypertension. The basic characteristics of the hypertensive participants are summarized in Table 1. There was a statistically significant difference in both systolic and diastolic blood pressure (P = 0.001), whereas other variables like gender, height, weight, and BMI showed no significant differences. The distribution of serum vitamin D levels among hypertensive patients is shown in Table 2. A statistically significant number of patients were found in the severely deficient category (Level I), indicating a potential association between low vitamin D levels and hypertension (p = 0.007).

## Discussion:

The association between vitamin - D levels and blood pressure (BP) has been explored in several studies, with many suggesting a negative correlation, where lower 25-hydroxyvitamin - D (25-OH vitamin - D) levels are associated with higher BP and increased prevalence of hypertension [8]. However, the evidence remains mixed and some studies fail to establish a significant link. Our study, in contrast, specifically focused on individuals with normal to high-normal BP in a disease-free cohort, which provides a novel perspective on the relationship between vitamin - D and BP in a relatively healthy population without existing cardiovascular comorbidities. Our findings show a strong inverse relationship between vitamin - D levels and BP, indicating that lower vitamin - D levels are associated with higher BP in hypertensive patients. This result aligns with studies like that of Jorde *et al.* who similarly found a significant association between vitamin - D deficiency and hypertension [9]. Furthermore, Sabanayagam *et al.* demonstrated that low vitamin - D levels were linked to prehypertension in U.S. adults, supporting the hypothesis that vitamin - D deficiency could play a crucial role in the development of hypertension from an early stage [10]. One of the most important mechanisms by which vitamin - D influences BP is its regulation of the renin-angiotensin-aldosterone system (RAAS). RAAS up regulation has long been recognized as a major risk factor for the development of hypertension [5, 6- 7]. Our study supports the idea that vitamin - D affects RAAS by binding to vitamin - D receptors (VDR) in renal and vascular tissues. In line with Forman *et al.* findings, we observed that lower 25-OH vitamin - D levels were associated with increased circulating levels of angiotensin II and impaired renal function in vitamin - D-deficient individuals [11]. Elevated angiotensin II contributes to increased vascular tone, fluid retention and consequently, higher BP. This can also lead to cardiac hypertrophy, a key feature of sustained hypertension.

Additionally, suppression of renin expression by vitamin - D is independent of its role in calcium metabolism and instead, it is linked to the body's regulation of sodium balance and angiotensin II feedback mechanisms [12]. This finding is corroborated by Li *et al.* [13] who demonstrated that vitamin - D receptor knockout mice exhibited higher renin levels, leading to increased angiotensin II and the development of hypertension and cardiac hypertrophy [7]. The role of vitamin - D in renin regulation is supported further by genetic studies, such as those by Vaidya *et al.* who found that polymorphisms in the vitamin - D receptor gene (Fok1) could increase plasma renin levels and predispose individuals to hypertension [14]. Beyond RAAS, insulin resistance is another important factor in the development of hypertension and vitamin - D deficiency has been linked to increased insulin resistance [15]. This metabolic dysfunction could further contribute to the pathogenesis of hypertension. Moreover, vitamin - D has been shown to inhibit vascular smooth muscle cell proliferation and a deficiency in vitamin - D can promote vascular remodeling, leading to arterial stiffness and the development of hypertension [16, 17-18]. Therefore, the lack of vitamin - D not only affects the vascular tone but also contributes to the structural changes in blood vessels that underlie elevated BP. While the antihypertensive effects of vitamin - D supplementation have been suggested by some studies, results have been inconsistent. Some research has failed to demonstrate significant reductions in BP with vitamin - D supplementation, raising important questions about optimal dosing, the duration of supplementation and the specific subpopulations that may benefit most. Interestingly, in our study, vitamin - D3 supplementation in obese hypertensive patients was found to modulate angiotensin II activity, similar to the effects of angiotensin-converting enzyme inhibitors, highlighting the complexity of vitamin - D's role in BP regulation [21, 22-23].

Additionally, parathyroid hormone (PTH), a key regulator of calcium homeostasis, is also involved in BP regulation. Secondary hyperparathyroidism, which is common in vitamin - D deficiency and primary hyperparathyroidism have both been associated with cardiovascular pathology, including hypertension. Our study found that higher PTH levels were significantly correlated with increased BP, consistent with prior reports indicating that PTH may elevate BP through multiple mechanisms. These mechanisms include increased renin release, endothelial dysfunction, arterial stiffness and activation of the sympathetic nervous system [19, 20, 24-25]. Our findings underscore the intertwined roles of vitamin - D and PTH in regulating BP, further emphasizing the importance of correcting vitamin - D deficiency in hypertensive patients. Moreover, the relationship between vitamin - D deficiency and metabolic syndrome is becoming increasingly evident. In our study, we found an inverse correlation between vitamin - D levels and several metabolic syndrome components, including increased BP, high triglycerides, low HDL, increased waist circumference and elevated fasting glucose [26]. Given that high-normal blood pressure (HNBP) is a key component of metabolic syndrome, our study suggests that vitamin - D deficiency may not only contribute to hypertension but also increase the risk of metabolic disturbances. This highlights the potential for vitamin - D supplementation to serve as a preventive strategy against both hypertension and metabolic syndrome. Our study employed the immunoassay method to measure serum 25-hydroxyvitamin - D (25-OH vitamin - D) levels, which is the standard clinical approach for assessing vitamin - D status. Although other metabolites, such as CYP11A1-derived secosteroids, play a role in vitamin - D metabolism, particularly in the skin and adrenal glands, serum 25-OH vitamin - D remains the most reliable indicator of overall vitamin - D status in clinical practice [27, 28, 29-30]. The results of the present study show that vitamin D deficiency is common in Urumqi, Xinjiang, China and vitamin D levels are negatively correlated with renin levels. Vitamin D plays an important role in regulating blood pressure by affecting renin levels through the renin-angiotensin-aldosterone system [31].

## Limitations:

The limitations of our study are a low number of patients and they are from a single center. The other limitation is population-based nature. The seasonal variation of vitamin - D may also affect its levels. Although Liquid Chromatography-tandem Mass Spectrometry method is the gold standard method for vitamin - D measurement now, we used the immunoassay method. Another possible limitation is the measurement of vitamin - D and hypertension at a single fixed time. Although 1.25-OH vitamin - D is the active metabolite, only 25-OH vitamin - D could be measured in this study. Dietary habits or the use of salt could not be standardized for the development of hypertension.

## Conclusion:

We show that serum 25-OH vitamin - D levels are independently associated with normal to high normal blood pressure and risk of incident hypertension in a middle-aged Bundelkhand Region population in Madhya Pradesh, India. The negative correlation between vitamin - D level and blood pressure in non- hypertensive individuals were found in our study. Further population studies with a large number of patients are needed to evaluate the role of serum 25-OH vitamin - D supplementation and/or level in preventing or delaying the development of hypertension in pre- hypertensive patients. Since vitamin - D values in our study group in Bundelkhand Region Madhya Pradesh, India were found to be very low; a community nutrition program should be developed to increase its level.

## Figures and Tables

**Table 1:** Basic characteristics of participants in this study

**S.N.**	**Variables**	**Category**	**Hypertensive group**		**P- Value**
			**Numbers**	**%**	
1	Gender	Male	72	72	0.91(c)
		Female	28	28	
2	Height	Mean	163.1cm	SD 6.4 cm	0.11(t)
3	Weight	Mean	65.9kg	SD 8.5 kg	0.1.(t)
4	BMI	Mean	25.1	SD 3.07	0.67(t)
5	Systolic BP	Mean	163	SD 16.7	0.001*(t)
6	Diastolic BP	Mean	95	SD 9.3	0.001*(t)
*p<0.05- The difference
between two groups is
statistically significant
c - Chi square test,
t - independent t test

**Table 2:** Serum vitamin - D levels in different categories

**Vitamin - D Levels**	**Number of patients**	**p- value**
Level-I (<37.5 nmol/L)	58	
Level-II (37.5- 49.9 nmol/L)	7	0.007*(c)
Level-III (50-74.9 nmol/L)	20	
Level-IV(>75 nmol/L)	15	
